# Minimal-Access Atrial Septal Defect (ASD) Closure

**DOI:** 10.3390/jcdd10050206

**Published:** 2023-05-10

**Authors:** Gillian Hardman, Joseph Zacharias

**Affiliations:** Department of Cardiothoracic Surgery, Blackpool Victoria Hospital, Blackpool FY3 8NR, UK

**Keywords:** minimal access, minimally invasive, cardiac surgery, atrial septal defect (ASD), congenital heart disease (CHD), adult congenital heart disease (ACHD), grown-up congenital heart disease (GUCH)

## Abstract

Progress towards the development and adoption of minimally invasive techniques in cardiac surgery has been slower than that seen in other surgical specialties. Congenital heart disease (CHD) patients represent an important population within cardiac disease, of which atrial septal defect (ASD) is one of the most common diagnoses. Management of ASD encompasses a range of minimal-access and minimally invasive approaches, including transcatheter device closure, mini-sternotomy, thoracotomy, video-assisted, endoscopic, and robotic approaches. In this article, we will discuss the pathophysiology of ASD, along with diagnosis, management, and indications for intervention. We will review the current evidence supporting minimally invasive and minimal-access surgical ASD closure in the adult and paediatric patient, highlighting peri-operative considerations and areas for further research.

## 1. Introduction

Minimal-access and minimally invasive approaches to congenital heart disease (CHD) have been adopted, with the development of percutaneous transcatheter device closure and a range of surgical approaches to septal defect closure. For the CHD patient, who is more likely to be younger, of working age or adolescent, the benefits of these approaches, with the potential for safe, effective treatment with good long-term outcomes, a reduction in trauma, fewer post-operative complications, shorter hospital stay, a faster return to functional status and improved cosmesis, cannot be underestimated.

The prevalence of CHD worldwide is approximately 9 per 1000 newborns [1], and while severe congenital heart defects are declining in many developed nations, globally, overall prevalence is increasing. With advances in technology, diagnosis and medical and surgical management, over the last decade, the majority of individuals born with CHD now survive into adulthood [2], with CHD representing a not-insignificant burden of disease in the cardiovascular disease patient population. CHD can be classified into mild, moderate or severe [3], with atrial septal defect (ASD) considered mild or moderate, depending on size, morphology and associated lesions. In this review, we will provide an overview of atrial septal defect pathology, diagnosis and management, including transcatheter and surgical approaches, with a focus on surgical intervention and the evidence base guiding current practice.

### 1.1. Atrial Septal Defect (ASD)

Atrial septal defect (ASD) is one of the most common congenital heart defects, accounting for 10% to 15% of all forms of congenital cardiac malformations [4]. ASD can remain undiagnosed into adulthood [3] with the majority of patients developing symptoms beyond the fourth decade.

Secundum ASD, located in the region of the fossa ovalis, is the most common defect type, accounting for approximately 80% of all ASDs. Primum ASD is synonymous with partial atrioventricular septal defect (AVSD), with communication at the atrial level only, and accounts for 15% of all ASDs. Superior sinus venosus defects (5% of ASDs) are located near the entry point of the superior vena cava (SVC) within the right atrium and are associated with the partial or complete connection of the right pulmonary veins to the right atrium. Inferior sinus venosus defects are located near the entry of the inferior vena cava (IVC) and account for less than 1% of atrial septal lesions. Finally, an unroofed coronary sinus represents a spectrum of anomalies where part or all of the wall between the coronary sinus and left atrium is absent. Most cases are associated with anomalous systemic venous return, including a persistent left superior vena cava, and represent the most rare form of ASD, at less than 1% [5]. The vascular anomalies and lesions most frequently associated with ASD include anomalous pulmonary venous connection, persistent left SVC, pulmonary valve stenosis, coarctation of the aorta and mitral valve prolapse. Conversely, ASD is frequently a component of other CHD lesions, including the Ebstein anomaly [3].

Pathophysiology is associated with the shunting of blood across the defect, with shunt volume dependent on the compliance of the left and right ventricles, the size of the defect and the pressure difference between the left and right atria. A simple ASD results in a left to right shunt because of the higher compliance of the right ventricle compared with the left ventricle, causing right ventricular volume overload and pulmonary over-circulation. A defect size of ≥10 mm is deemed to result in a clinically relevant shunt [3]. Any condition resulting in a reduction in left ventricular compliance or elevation of left atrial pressure, for example, systemic hypertension, ischaemic heart disease, cardiomyopathy or left sided valve lesions (aortic and mitral valve disease) will result in an increase in the left to right shunt. For this reason, an ASD may become more clinically and haemodynamically important with increasing age. Conversely, conditions that decrease right ventricular compliance will reduce the left to right shunt and may eventually cause shunt reversal (Eisenmenger syndrome), with resultant cyanosis [6].

Most commonly, patients develop symptoms associated with reduced functional capacity, including shortness of breath on exertion, palpitations (associated with supraventricular tachyarrhythmias) and less frequently, pulmonary infection and right heart failure. Life expectancy is reduced overall, but survival is better than previously assumed [7]. Pulmonary artery pressure can be normal but typically increases with age in the un-repaired ASD. With increasing age and increasing pulmonary artery pressure, tachyarrhythmias, for example, atrial fibrillation (AF) and atrial flutter, become more common [8]. Systemic embolism may occur due to AF or atrial flutter or, more rarely, paradoxical embolism, which may prompt investigation for ASD in the previously undiagnosed, asymptomatic adult. Clinical findings include fixed splitting of the second heart sound and a systolic pulmonary flow murmur. Electrocardiogram (ECG) classically shows a partial right bundle branch block with right-axis deviation, and an increased pulmonary vascularity may be noted on a plain chest radiograph [3]. Echocardiography is the first-line diagnostic technique.

#### ASD Closure

The diagnosis and management of ASD in the adult is outlined in the 2020 ESC Guidelines for the management of adult congenital heart disease [3]. Indications for ASD closure include evidence of right ventricular volume overload, and in the absence of pulmonary hypertension (PH) and left ventricular disease, ASD closure is recommended as a Class I, level B indication, regardless of symptoms. In patents with suspicion of paradoxical embolism, ASD closure should be considered, regardless of size, providing there is absence of PH and left ventricular disease (Class IIa, level C).

Percutaneous transcatheter device closure is considered the first-choice treatment for most ASDs however surgical repair is indicated for limited non-secundum ASD, or secundum ASD characterised by large defects, insufficient septal rim or a left atrium that is too small to accommodate a closure device [9]. Surgical repair has low mortality (less than 1% in patients without significant comorbidity) and good long-term outcomes when performed in adolescence and early adulthood, and in the absence of pulmonary hypertension [10,11]. With increasing patient age, surgical ASD repair can still be considered, with low operative risk, however comorbidities may affect operative risk and should be weighed against the potential benefit.

### 1.2. Device Closure

Device closure for cardiac septal defects (including ASD and ventricular septal defect (VSD)) was developed in the 1990s, with the deployment of the device performed either percutaneously or by mini-thoracotomy approaches. The advantages of transcatheter device closure include less trauma, faster post-procedure recovery and improved cosmesis. Device closure is recommended for the treatment of secundum ASDs, when technically feasible (Class I, level C). Feasibility for device closure depends on the morphology of the defect and includes a stretched diameter ≤38 mm, with a sufficient rim of remaining septum of 5 mm, except towards the aorta [3]. Several studies have reported no mortality following device closure, and serious complications have been observed in less than 1% of cases [12,13]. Complications of device closure include early atrial tachyarrhythmias and, more rarely, erosion of the atrial wall, anterior mitral leaflet or the aorta, as well as thromboembolic events [14,15]. Antiplatelet therapy (a suggested minimum treatment with Aspirin 75 mg once daily) is required for at least 6 months following the procedure. Studies comparing transcatheter intervention and surgical ASD closure have reported similar survival rates. Hospital length of stay and procedure-related complications are lower in the in the catheter intervention group but with slightly higher rates of residual shunt and reintervention compared to the surgical group [12,16,17,18].

### 1.3. Surgical Closure

Surgical ASD closure has been previously reviewed [19], and repair via median sternotomy, using cardiopulmonary bypass (CPB) instituted with the cannulation of the ascending aorta and both vena cavae, is considered the standard approach to surgical treatment. For isolated secundum defects, this approach can be performed with excellent results and mortality approaching zero percent [20]. Complications of surgical ASD repair include arrhythmia, pneumothorax, bleeding, pericardial and pleural effusions. As outlined above, the consequences of post-operative complications will be more marked with increasing patient age and comorbidity and with arrhythmia and prolonged intensive care unit (ICU) and hospital stay, and they are more common in adults and older patients [21]. When surgery is performed in the young adult, the long-term results for surgical secundum defect-closure return actuarial survival curves similar to that of the general population [10].

The American Heart Association defined “minimally invasive” as smaller sternotomy or non-sternotomy strategies aided by robotic or video-assisted technologies [22], and a variety of approaches have been described. Access route is a minor component of invasiveness within cardiac surgery, with cardiopulmonary bypass (CPB), aortic cross clamping, cardioplegic arrest and opening of cardiac chambers contributing more to the invasiveness and risk of complications of the procedure, beyond that of the incision [23]. For the surgical treatment of ASD, minimal-access approaches include partial or mini-sternotomy, right anterolateral thoracotomy, right oblique sub-axillary incision, right vertical sub-axillary incision, right posterior mini-thoracotomy, video-assisted thoracoscopy (VATS)/endoscopic and robotic approaches, with peripheral cannulation for cardiopulmonary bypass. Multiple heart centres have reported their experiences of minimal-access surgical ASD closure for both paediatric and adult congenital populations worldwide.

#### 1.3.1. Minimal-Access Surgery versus Median Sternotomy

**Figure 1:**
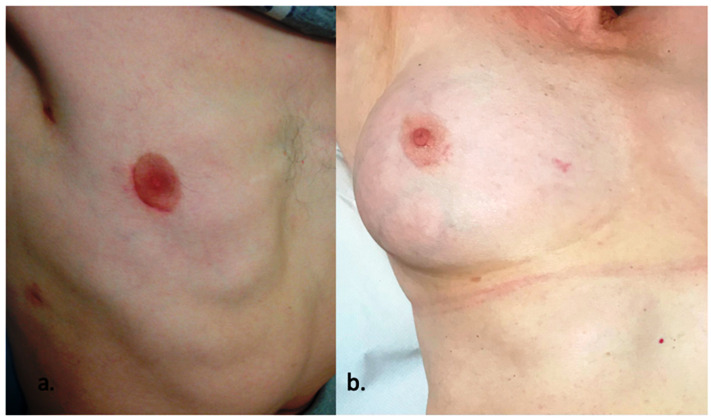
Healed periareolar scars in (**a**) a male patient and (**b**) a female patient 4 months after endoscopic atrial septal defect closure.

The mini-sternotomy approach to CHD has been studied, with an associated reduction in post-operative drainage, shorter hospital length of stay and improved cosmesis compared to standard median sternotomy [24]. Right anterolateral mini-thoracotomy has been widely applied as an alternative to median sternotomy, with similar mortality and post-operative morbidity and superior cosmetic results compared to sternotomy. Minimally invasive video-assisted and endoscopic surgical closure have previously been described with detailed outlines of the approach and surgical technique [25,26]. Single-centre reports describing the evolution of practice and decades-long experience with minimal-access approaches to ASD closure in the adult have been reported [27,28,29,30,31], including comparisons of median sternotomy, totally thoracoscopic and axillary thoracotomy access techniques [32]. A recent systematic review and meta-analysis by Lei et al. [33], including 7 publications and 665 patients, compared short-term outcomes between anterolateral mini-thoracotomy and median sternotomy for the surgical treatment of ASD. They concluded that both approaches were equally safe and effective, with similar success and complication rates. Anterolateral mini-thoracotomy was associated with a faster return to function and better cosmetic results compared to the sternotomy group. The cosmetic benefit is amplified with the use of the periareolar incision, which is adopted within our unit, and it is shown in Figure 1.

As outlined within the guidelines for ASD management, surgical closure is becoming increasingly relevant, compared to transcatheter device closures, for more-complex atrial septal lesions. Clinical case series comparing minimal-access approaches with median sternotomy for adult and paediatric patients with sinus venosus defects and partial anomalous pulmonary venous drainage [34,35], for the treatment of unroofed coronary sinus [36] and more-complex grown-up congenital heart disease [37], have been reported. Although the proportion of patients undergoing minimal-access approaches is relatively small, all conclude that this approach is as safe and effective as conventional median sternotomy.

#### 1.3.2. Minimal-Access Surgery versus Transcatheter Device Closure

A comparison between transcatheter device closure and minimal-access surgical closure for ASD has been made. In single-centre retrospective reviews for secundum ASD closure in adults, a minimally invasive approach was found to achieve a more complete closure compared to transcatheter device closure methods, along with decreased rates of AF and anticoagulation use [38], with another study identifying zero mortality and similar rates of serious complications in both groups [39]. More recently, Goh et al. [40] performed a systematic review and meta-analysis comparing minimally invasive and transcatheter approaches for secundum ASD repair, which included 6 observational studies and a total of 1524 patients. As with the median sternotomy approach, they highlighted that transcatheter closure was associated with shorter hospital length of stay and lower rates of pneumothorax and pericardial effusion, but with higher rates of residual shunt. They concluded that minimally invasive repair had similar outcomes to device closure but recognised the need for further randomised controlled trials comparing the two. Similar meta-analyses have also been reported within the paediatric literature [41]. Finally, a single-centre experience comparing transcatheter device closure and totally endoscopic robotic closure has described similar findings, with shorter hospital and ICU length of stay for the transcatheter group but similar complication risk profiles between the two groups [42].

## 2. Insights

### 2.1. Pre-Operative Work-Up and Patient Selection

Echocardiography is the first-line technique in the diagnosis and quantification of ASD. Right ventricular volume overload, which may be the first unexpected finding in a patient with previously undiagnosed ASD, is the key finding and best characterises the haemodynamic relevance of the defect. In general, sinus venosus defects require trans-oesophageal echo (TOE) for accurate diagnosis, with cardiovascular magnetic resonance imaging (CMR) or cardiovascular computed tomography (CCT) alternative modalities in cases of inferior sinus venosus defects. TOE is required for the precise evaluation of secundum defects before device closure, where assessment includes sizing, exploration of the residual septum’s morphology, rim size and quality, exclusion of additional defects and confirmation of normal pulmonary venous connection. Other key information includes the estimation of pulmonary artery pressure (PAP) and tricuspid regurgitation (TR).

CMR has become an essential modality in specialist units for the assessment of complex CHD, enabling 3D anatomical reconstruction, which is not restricted by body size or acoustic windows and avoids exposure to radiation [43]. Of note, adults with CHD with conventional pacemakers and defibrillators can undergo CMR where local support is available [44]. CMR is rarely required in the diagnosis and pre-operative planning of ASD repair but may be useful for the assessment of RV volume overload, identification of inferior sinus venosus defects, quantification of pulmonary to systemic flow ratio (Qp:Qs) and evaluation of pulmonary venous connection. CCT can be used to assess ventricular size and function, with inferior temporal resolution compared to CMR and typically higher radiation dose. CCT does, however, benefit MICS ASD repair assessment through high spatial resolution and rapid acquisition time, with the ability to aid pre-operative planning with the assessment of the systemic great vessels, coronary arteries, collateral arterial supply and lung parenchyma. Developments have substantially reduced the amount of radiation exposure for combined CCT coronary, pulmonary and aortic angiograms, making CCT a more attractive option for pre-operative planning in ACHD patients [45].

Cardiac catheterisation is required in the case of non-invasive signs of raised pulmonary artery pressure (PAP) and is used to determine pulmonary vascular resistance (PVR). Pulmonary hypertension (PH) is an important prognostic factor in CHD. PH is now defined as an increase in invasively measured PAP ≥ 20 mmHg at rest [46]. Pulmonary hypertension in ASD (and other shunt lesions) typically manifests as pre-capillary PH (PAH), defined as a mean PAP > 20 mmHg, with a pulmonary arterial wedge pressure (PAWP) of ≤15 mmHg and a pulmonary vascular resistance of ≥3 woods units (WU). The successful management of an ACHD patient with PH requires a multidisciplinary team approach, and where PH is identified, exercise testing should be performed to exclude desaturation. Cardiopulmonary exercise testing (CPET) provides an evaluation of functional capacity and physical fitness prior to surgery, which correlate well with morbidity and mortality in ACHD patients [47]. Pulmonary Function Tests (PFTs) can also be useful in the evaluation of patients for MCS, where single-lung ventilation is planned, in the context of known respiratory disease or significant positive smoking history.

### 2.2. Anaesthetic Considerations

A recent review of anaesthesia for minimally invasive cardiac surgery has been published [48] outlining the anaesthetic considerations common to all minimally invasive cardiac surgical procedures.

Beyond mini-sternotomy, all other access for surgical ASD closure requires single-lung ventilation prior to cardio-pulmonary bypass, to facilitate right mini-thoracotomy and the placement of utility ports. This can be achieved using a double lumen endotracheal intubation or a single lumen tube with intermittent ventilation or an endobronchial blocker.

To establish cardiopulmonary bypass in the context of right atriotomy for ASD closure, the inferior vena cava (IVA) is cannulated by the surgeon by insertion into the femoral vein, advancing distal to the cavoatrial junction using TOE guidance from the anaesthetist. The SVC cannula is placed in the right internal jugular vein via the Seldinger technique and advanced proximally during anaesthetic induction, using ultrasound guidance. Alternative peripheral perfusion strategies for cardiopulmonary bypass in ASD closure have been reported from a single-centre, retrospective review [49].

As discussed above, patients with raised pulmonary artery pressures and pulmonary hypertension must be carefully evaluated. In patients with PVR < 5 woods units, ASD closure has been shown to be safe and associated with a decrease in PAP and an improvement of symptoms [50,51,52]. Patients with PVR ≥ 5 woods units are unlikely to improve [52] and more likely to have worse outcomes with complete ASD closure [53]. In patients with impaired left ventricular function (systolic and diastolic), ASD closure may worsen heart failure. Again, careful consideration must be paid to pre-operative evaluation and peri-/post-operative haemodynamic inotropic management. Pre-interventional testing (balloon occlusion with reassessment of haemodynamics) can aid operative decision making with the potential for complete, fenestrated or no closure, considering that an increase in filling pressure due to the ASD closure may worsen symptoms and outcome [54].

### 2.3. Surgical Technique

**Figure 2:**
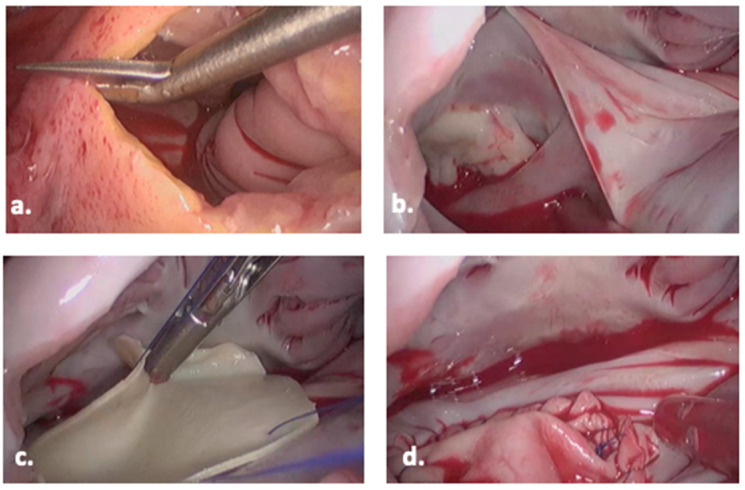
Operative view of pericardial patch closure of secundum ASD: (**a**) the right atrium is opened, (**b**) the boundaries of the ASD are identified, and (**c**) sutures are placed in the rim of the ASD and bovine pericardium is parachuted into the right atrium. The ASD repair is continued using a running suture and (**d**) the patch repair is complete.

The surgical technique, including operating room set-up, positioning, establishing cardiopulmonary bypass and pericardial patch closure, for minimally invasive ASD repair via right mini-thoracotomy, is well described by Nagendran et al. [26] and mirrors the approach taken within our centre. Approaches to myocardial protection during minimally invasive mitral valve surgery have been summarized by Garbade et al. [55] and are applicable to minimally invasive ASD repair. Intra-operative views of the endoscopic right mini-thoracotomy pericardial patch ASD repair are shown in Figure 2.

Beyond these technical considerations, specific issues relating to minimal-access surgical ASD repair include the persistent left SVC, anomalous pulmonary venous connections, and other associated anomalies, including valve lesions and atrial arrhythmias. In patients with atrial flutter/AF, cryo- or radio-frequency ablation (modified maze procedure) should be considered at the time of surgery [3]. Here, surgical ASD closure benefits the patient, as device closure may restrict access to the left atrium and limit the potential for electro-physiology (EP) interventions at a later date. Evidence for the surgical or interventional treatment of adult patients with ASD and AF suggests that there is a reduction in the prevalence of AF after ASD closure alone. This is more likely for paroxysmal AF but less successful for persistent or long-standing AF. The reduction in recurrence rate for AF, post-ablation, is highest with bi-atrial surgical ablation [56]. For the patient with persistent AF in the context of ASD, these factors may influence decision making and surgical approach.

Anomalous pulmonary venous connections do not only occur in association with ASD (typically sinus venosus defects) but can also be isolated. Pathophysiologically, these connections result in volume overload of the right heart, with a physiological effect similar to that of an ASD. Most common are the connection of the right pulmonary vein(s) to the IVC (“scimitar vein”, which might be associated with sequestration of the right lower lobe), the left upper pulmonary vein(s) to the left innominate vein, and the right upper pulmonary vein(s) connecting high on the SVC. A case report of an anomalous left hepatic vein to coronary sinus in a patient with ASD is also reported [57]. Here, the anomalous connection was repaired using a minimally invasive approach. Surgical repair can be challenging, and low-velocity venous flow imparts the risk of thrombosis of the surgically operated vein, particularly in scimitar syndrome. Indications for surgery follow the principles of recommendations for ASD closure, but technical suitability for the repair and operative risk must be weighed against the potential benefit of intervention [3]. Anomalous venous connections and systemic vascular anomalies can prove technically challenging within minimal-access surgery due to the inability to maintain adequate venous drainage, flooding of the operative field and ventricular distension. These vascular anomalies should be identified pre-operatively, with a careful review of imaging and a high index of suspicion in appropriate patients.

### 2.4. Post-Operative Care

Immediate post-operative care of the minimal-access ASD closure patient does not differ significantly from that of the adult patient undergoing standard sternotomy closure. After weaning from CPB, the pericardium is loosely closed with the placement of a small pericardial drain. A second drain is placed in the right pleura. Following transfer to the cardiac ICU, a post-operative chest radiograph is performed to ensure right lung expansion and confirm the position of the right pleural drain. Drain removal typically occurs within 24 to 48 h post-surgery, once drainage has ceased and residual pneumothorax is excluded.

As per ESC and AHA guidelines, evaluation following ASD closure is the same, regardless of closure method, and should include an assessment of residual shunt, RV size and function, presence of tricuspid regurgitation and PAP measurement using echocardiography. Patients should be assessed for the presence of arrhythmias by history, ECG and, if suspected, Holter monitor. For patients who undergo ASD closure aged over 40 years, the prevalence of atrial arrhythmias is up to 60%. Anticoagulation should be considered for patients with persisting atrial arrhythmias, and in the absence of arrhythmia, antiplatelet medication can be considered for at least six months following surgical repair, although guidance around dose and duration of treatment is limited. Patients repaired below the age of 25 years, without relevant sequelae or residual disease (no residual shunt, normal PAP, normal right ventricular volume and function, no arrhythmias) do not require regular follow-up. However, patients should be informed of the possibility of late-occurrence tachyarrhythmias. Patients with residual shunt, elevated PAP or arrhythmias (before or after repair) and those repaired at adult age (particularly >40 years) should be followed up on a regular basis [3].

As with the adult congenital population more widely, specific guidance should be given to those patients following ASD closure, with regards to future pregnancy, which, in the absence of pulmonary hypertension, can be considered low risk in this patient population.

The risk of infective endocarditis (IE) in ACHD patients is higher than that of the general population, with marked variation between lesions. The 2015 ESC Guidelines on IE maintain that antibiotic prophylaxis for invasive procedures should be restricted to the highest-risk patients for the highest-risk procedures [58]. High-risk conditions include any CHD repaired with prosthetic material, up to 6 months after the procedure, or lifelong if residual shunt or valvular regurgitation remains. All un-repaired and repaired ACHD patients should be counselled around good oral and cutaneous hygiene, aseptic measures during invasive procedures, the avoidance of unnecessary invasive procedures, such as piercings or tattoos and the signs and symptoms of IE, along with the promotion of health-seeking behaviour during any episodes of infection.

## 3. Robotic ASD Closure

As minimal-access and minimally invasive cardiac surgical techniques advance, the role for robotic surgery is increasingly considered. The use of robotic surgery for ASD closure has been reported for a cohort of 54 adult and paediatric cases with secundum ASD using the da Vinci robotic system, with the cannulation of the femoral vessels and right internal jugular vein [59]. As with all developing techniques, significant learning curves were reported, evidenced by cross-clamp time, cardiopulmonary bypass time and operative duration, with the authors concluding that ASD closure can be safely and effective performed using a totally endoscopic approach and robotic surgery. More recently, robotically assisted congenital cardiac surgery has been retrospectively reviewed in a single centre, including 242 procedures for secundum ASD, sinus venosus ASD, partial anomalous pulmonary venous drainage and unroofed coronary sinus, as well as other CHDs, in adult patients. Conversion to larger thoracic incision was required in 0.8% of cases, and a mean hospital stay of 3.5 (±1.1) days was reported [60].

### 3.1. Minimal-Access Surgery and the Paediatric Patient

Although the focus of this review is on the adult ASD population, minimal-access surgical approaches to ASD closure in the paediatric population have been reported. The development of transcatheter device closure in children is well described [61], and the use of lower mini-sternotomy approaches to surgical management, in order to improve cosmesis, are also reported [62]. The range of minimal-access approaches to ASD closure in the paediatric population, including details of the operative approach and technical considerations, has previously been reviewed [63]. In 2011, Wang et al. reported on 28 patients, with a mean age of 5.8 years and a mean weight of 15 kg, undergoing totally thoracoscopic surgery for ASD closure, with no mortality or re-intervention and of New York Heart Association (NYHA) functional class I, at 6-month follow-up [64]. Similar outcomes have since been described by other groups [65], with Sabzi et al. comparing conventional median sternotomy and modified anterior mini-thoracotomy in 54 children [66]. Here, again, the benefit of a minimal-access approach to surgical closure compared to sternotomy for more-complex ASDs was emphasised.

### 3.2. Patient Perspectives

In contrast to general surgery, where laparoscopic approaches have been widely and rapidly implemented, the adoption of minimal-access approaches to cardiac surgery have been much slower. As highlighted in the evidence presented within this review, median sternotomy is a well-tolerated incision, and the invasiveness of a cardiac surgical procedure extends beyond that of access alone. With high success rates and low procedural risk and mortality across all approaches to ASD closure, including transcatheter devices, conventional median sternotomy and minimal-access surgical approaches, the assessment of other patient-related outcome measures takes on increasing significance. Within the paediatric population, health-related quality of life (HRQL) has been compared for children undergoing interventional closure and minimally invasive surgical closure for both ASD and VSD repair [67]. The group concluded that HRQL continuously improved post-procedure, regardless of the type of intervention; however, catheter-based intervention was associated with better HRQL in the early post-procedure period.

Patient reported outcome measures (PROMS) and patient reported experience measures (PREMS) are standardised tools used to collect data about the subjective assessment of medical care from the perspective of the patient and are increasingly implemented across of range of health care settings. A recent letter from Kent [68] summarises the importance of qualitative outcome measures in the assessment of minimally invasive techniques, including measures such as pain scores, functional disability and return to daily activities. These factors are likely to be a priority to patients, beyond mortality and post-operative complications, particularly when a range of safe and effective management options are available to them. The ability to adequately describe the impact of a procedure for a given patient is fundamental to informed consent and shared decision making. Within minimally invasive robotic cardiac surgery, across a range of procedures, the study of patient body image, self-esteem and cosmetic result identified benefits in comparison to conventional sternotomy [69]. For the CHD population, who are younger and more likely to be in adolescence or of working age, these factors potentially take on increased significance.

### 3.3. Team Working and Safe Implementation

The importance of a heart team approach to the management of ASD, and ACHD more widely, should not be underestimated. With the range of approaches to ASD closure all proving to be safe, effective and with low to minimal risk, individualised patient decision making, including shared decision-making models based on the patients’ priorities, should be implemented, particularly as expertise and volume for minimal-access approaches grow. For all minimally invasive cardiac surgical programmes more generally, widespread implementation requires careful consideration and a team-based approach to ensure the safe delivery of care, including adequate training for the operative team, provision of post-operative care and patient follow-up. This approach has recently been highlighted in the UK [70] and has been described in our own centre [71].

## 4. Conclusions

A minimal-access surgical approach to closure of atrial septal defects should be considered for all patients discussed at an ACHD multi-disciplinary team meeting. Patients do benefit from this approach when it is performed in experienced centres, and the advantages extend to both the patient and the institution. Access to minimal-access and minimally invasive ASD closure likely requires increased collaboration between ACHD centres and cardiac surgical centres with expertise in endoscopic or robotic cardiac surgery procedures. We hope this review gives confidence to both physicians and patients considering the surgical closure of an ASD to find an experienced team to offer a minimally invasive, cosmetically superior option.
